# Characterization of the Diversity in Host Range of an Extensively Drug-Resistant (XDR) Type IV Secretion System-Encoding Plasmid in *Acinetobacter*

**DOI:** 10.3390/pathogens14060606

**Published:** 2025-06-19

**Authors:** Kailey Martz, Dalya Alomar, Marisha Karim, Sara Knezevic, Vanessa M. D’Costa

**Affiliations:** 1 Department of Biochemistry, Microbiology and Immunology, University of Ottawa, Ottawa, ON K1H 8M5, Canada; 2 Centre for Infection, Immunity and Inflammation, University of Ottawa, Ottawa, ON K1H 8M5, Canada

**Keywords:** *Acinetobacter baumannii*, bacterial pathogen, antibiotic resistance, carbapenem resistance, conjugation, horizontal gene transfer, secretion system

## Abstract

The World Health Organization (WHO) cites antimicrobial resistance as among the greatest threats to human health. The multidrug-resistant pathogen *Acinetobacter baumannii*, recognized as a priority pathogen for healthcare and research, is responsible for a diverse array of infections including respiratory tract, soft tissue and wound, and bloodstream infections. Despite this importance, the mechanisms of its pathogenesis remain poorly understood. Conjugation represents a central mechanism for bacterial adaptation and evolution and is responsible for the spread of genes that promote pathogen survival, antibiotic resistance, virulence, and biofilm formation. Our laboratory recently characterized a large group of almost 120 Type IV Secretion System (T4SS)-encoding plasmids in *Acinetobacter*, distributed globally across 20 countries spanning four continents, and demonstrated that an XDR *A. baumannii* plasmid from this family was transmissible to another *A. baumannii* strain. This research investigated the potential diversity of host strains for this representative member plasmid. Using the GC1 lineage strain *A. baumannii* AB5075-UW harbouring the XDR plasmid p1AB5075 and a series of previously characterized clinical and environmental *Acinetobacter* strains, conjugative analyses demonstrated transfer of the XDR plasmid to both *A. baumannii* strains of more genetically divergent sequence types and to non-*baumannii Acinetobacter* species both inside and outside the *Acinetobacter calcoaceticus–baumannii* (ACB) complex. Successful recipients included diverse strains of both clinical and environmental origin within the *Acinetobacter* genus. Collectively, this research could provide insights into an important genetic element for future surveillance.

## 1. Introduction

The World Health Organization (WHO) has cited antimicrobial resistance (AMR) as one of the greatest threats to human health globally [1]. Often conferring resistance to front-line antimicrobials such as carbapenems and cephalosporins, the multidrug-resistant (MDR) bacterium *Acinetobacter baumannii* has been recognized by the WHO as a priority pathogen for the healthcare and research communities [2,3]. *A. baumannii* is responsible for a diverse array of infections including respiratory tract, soft tissue and wound, and bloodstream infections [2,3]. This hospital-acquired Gram-negative pathogen is a common cause of opportunistic infections in hospital burn wards and intensive care units (ICUs) [4]. *A. baumannii* has been estimated to account for over 20% of nosocomial bacterial infections globally [5]. Nosocomial *A. baumannii* is commonly acquired from hospital ventilators, where it can survive clinical disinfection protocols [6], and is estimated to represent up to 50% of ventilator-associated bacterial pneumonia cases [7]. Outside healthcare settings, increasing incidences of community-acquired *A. baumannii* infection have also been observed [2,8]. Exacerbating this challenge, horizontal gene transfer is acknowledged to play an important role in pathogen evolution for *A. baumannii* [4], contributing to the emergence of extensively drug-resistant (XDR) and pandrug resistant (PDR) strains globally [4,9,10,11].

*A. baumannii* is classified as a member of the *Acinetobacter calcoaceticus–baumannii* (ACB) complex, a group of clinically important species that includes *A. baumannii*, *A. calcoaceticus*, *A. pittii*, *A. nosocomialis*, *A. seifertii*, and *A. dijkshoorniae* [12,13]. *Acinetobacter* is a phylogenetically diverse genus, with species including *A. baumannii* capable of occupying diverse ecological niches, from human-host-associated settings to aquatic, soil, and plant-associated ecosystems [14,15,16,17,18,19]. Additional species of clinical importance outside the ACB complex include *A. haemolyticus*, *A. junni*, *A. lwoffi,* and *A. johnsonii* [14,20]. Among these, a number of species are considered to be emerging human pathogens [21,22,23,24].

While antibiotic resistance has been recognized as a global challenge with *A. baumannii*, resistance in clinically relevant non-*baumannii* species of *Acinetobacter* is also a growing concern [25,26]. Similarly, antibiotic resistance, including MDR phenotypes, has been described in diverse environmentally-derived *Acinetobacter* [27,28,29]. In the context of clinically important drugs, resistance to carbapenems, cephalosporins, and aminoglycosides such as tobramycin has been characterized in non-*baumannii* species of *Acinetobacter* of both clinical and environmental origin [27,28,29,30,31,32,33]. Given the importance of maintaining a One Health perspective to understanding this global challenge, it is imperative to maintain an integrated approach to antimicrobial resistance research [34].

Bacterial conjugation is an important driver of both genome diversification and bacterial adaptation [35]. Representing a central mechanism of horizontal gene transfer in bacteria, conjugation mediates the transfer of DNA from donor to recipient strains via a multi-subunit protein apparatus [35]. Conjugation plays an intrinsic role in pathogen evolution, promoting the spread of genes that mediate antibiotic resistance, pathogen survival, virulence and biofilm formation [35]. Conjugation in *Acinetobacter* has been shown to mediate the spread of one or more resistance genes from strains of both clinical and agricultural importance [28,36,37,38,39,40,41,42,43,44].

While conjugative plasmid transfer is better understood in other microorganisms, the mechanisms of this process are less understood in *Acinetobacter* [45]. A family of plasmids encoding a F-type Type IV Secretion System (T4SS) with genetic similarity to the F-plasmid in *Escherichia coli* was previously identified in *A. baumannii*, spanning a number of clinically important lineages [46,47,48]. This group of plasmids included a large subset in multilocus sequence type (MLST) ST-2 and ST-1 strains (Pasteur scheme), such as global clone II (GC2) epidemic MDR strain *A. baumannii* ACICU and global clone I (GC1) XDR strain *A. baumannii* AB5075-UW [46,47,48,49].

Recently, our laboratory examined the genetic diversity and geographical distribution of these T4SS-encoding plasmids [37]. This systematic analysis found that the plasmids were globally distributed and more widely spread across *A. baumannii* and the *Acinetobacter* genus than previously anticipated [37]. The large group consisted of almost 120 members distributed globally across 20 countries spanning four continents in strains of both clinical and environmental origin [37]. Phylogenetically, plasmids were identified in a broader range of *Acinetobacter* than previously anticipated, including 13 MLSTs of *A. baumannii* and 4 established species of *Acinetobacter* [37]. Of importance, this study identified an abundance and diversity of antibiotic resistance determinants on these plasmids, with almost half of plasmids harbouring resistance determinants and 16 different genes identified across the plasmids [37].

The XDR plasmid p1AB5075 from *A. baumannii* AB5075-UW is a member of this family [49]. *A. baumannii* AB5075-UW has been established to be highly virulent and is considered a model strain for pathogenesis research [49,50]. The 83.6 kbp plasmid p1AB5075 harbours 11 different antibiotic resistance determinants (*sul1*, *qacEΔ1*, *dfrA7*, *aac(6′)-Ib10*, *bla_GES-11_*, *aph(6)-Id*, *aph(3″)-Ib*, *aadA2*, *cmlA1*, *ant(2″)-Ia*, and *aph(3′)-VIa*), collectively encoding resistance to five different classes of antibiotics [49]. The T4SS-encoding gene cluster contains the putative genes *traT*, *traD*, *traI*, *traA*, *traL*, *traE*, *traK*, *traB*, *trbG*, *traV*, *traC*, *traW*, *traU*, *trbC*, *traN*, *traF*, *traH*, and *traG* [51]. In the context of mobile genetic elements, the plasmid contains elements TnAs3, Tn5393, and ISAba125 [49]. Plasmid p1AB5075 was previously used as a representative member of the F-type T4SS-encoding plasmid family and was shown to be capable of transmission to a more genetically divergent *A. baumannii* sequence type [37]. Given the diverse antibiotic resistome harboured on these plasmids and the importance of maintaining a One Health perspective to AMR research [34], their capacity for transmission within the broader *Acinetobacter* genus is of importance. Here, we examined the capacity for the transmission of this XDR T4SS-encoding plasmid to more divergent environmental isolates of *A. baumannii*, as well as more genetically divergent *Acinetobacter* strains of both clinical and environmental origin.

## 2. Materials and Methods

### 2.1. Bacterial Strains and Culture Conditions

*A. seifertii* CIP 110471, *A. haemolyticus* ATCC 17906, and *A. junii* ATCC 17908 were obtained from DSMZ, and *A. baylyi* ATCC 33305 was obtained from ATCC. *A. nosocomialis* DSM 102856, and *A. baumannii* AB046, AB047, AB048, AB052, and AB053 were provided as a kind gift from Dr. Ayush Kumar [52]. *A. baumannii* AB5075-UW was obtained from BEI Resources. This isolate harbours the T4SS-encoding plasmid p1AB5075 [49] and served as a conjugation donor strain [37]. Recipient strains harboured pMQ715 [51], kindly provided as a gift from Dr. Robert Shanks, to confer tetracycline-resistance for conjugation assays [37,53,54]. *Micrococcus luteus* GDW1580 was provided as a kind gift from Dr. Gerard Wright.

All bacterial strains were cultured according to previously documented culture conditions. *A. baumannii* AB5075-UW was cultured in LB Broth (Miller) (BioShop, Burlington, ON, Canada). *A. nosocomialis* DSM 102856, *A. seifertii* CIP 110471, and *A. baylyi* ATCC 33305 were grown in Tryptic Soy Broth (TSB) (BD, Mississauga, ON, Canada). *A. haemolyticus* ATCC 17906, *A. junii* ATCC 17908, and *A. baumannii* AB046, AB047, AB048, AB052 and AB053 were cultured in Nutrient Broth (NB) (BD, Mississauga, ON, Canada). All *A. baumannii* strains and *A. seifertii* CIP 110471 were cultured at 37 °C, and all other *Acinetobacter* strains were grown at 30 °C. *Micrococcus luteus* GDW1580 was grown on Tryptic Soy Agar (TSA). Unless specified otherwise, when appropriate, media were supplemented with the following antibiotics: kanamycin (50 μg/mL) (BioShop, Burlington, ON, Canada), ciprofloxacin (8 μg/mL) (MilliporeSigma, Oakville, ON, Canada), and tetracycline (10 or 30 μg/mL) (BioShop, Burlington, ON, Canada).

### 2.2. Conjugation Assays

Conjugation assays were performed using a previously described method [37,54]. Briefly, donor and recipient cultures were grown in the appropriate medium overnight at 250 rpm and either 30 °C or 37 °C. Cultures were supplemented with the appropriate antibiotics (kanamycin and ciprofloxacin for the donor; tetracycline for recipient). The donor was subcultured and grown to the logarithmic phase. Following washing with culture medium twice, cells were resuspended to an OD_600_ of 1.0. Donor and recipient cells were mixed at a ratio of 1:1 and plated on agar medium in the absence of antibiotics overnight. The growth medium and temperature associated with the recipient was used, as the donor strain was previously observed to grow comparably on all culture media and at all temperatures. Cells were resuspended in culture medium, and this resuspension was used to both quantify the conjugation frequency and select for transconjugants. For quantifications, serial dilutions of the resuspension were performed and plated onto agar medium supplemented with the appropriate antibiotic for subsequent colony forming unit (CFU) analysis. Conjugation frequencies were quantified as the number of transconjugants per recipient [36,55]. To select for transconjugants, the remaining resuspension was concentrated and plated onto the appropriate agar medium supplemented with kanamycin and tetracycline. Candidate transconjugants were assessed by replica plating onto agar medium with and without each antibiotic. For each recipient, three biological replicate conjugation assays were performed. Where applicable, strains were also replica plated onto media containing trimethoprim (64 μg/mL) or sulfamethoxazole (64 μg/mL).

### 2.3. Characterization of Transconjugants

To confirm transconjugant identity, strains were assessed for both the genetic background and the presence of plasmid p1AB5075. For typing of the genetic background for *A. baumannii* strains, an assessment of MLST genes was carried out using a previously established approach using primers for the housekeeping genes *rplB* or *fusA* [56]. For genetic background typing in non-*baumannii Acinetobacter* strains, 16S rRNA was assessed using previously established primers [57].

To assess the plasmids harboured by candidate transconjugants, a series of diagnostic PCRs were performed using previously established primers [37]. The T4SS primer set (5′-AAAGGCTCAGATCCAGAACAAGTA-3′ and 5′-TGCAAAAGCTAAAATACCATC-AAC-3′) was used for amplification of the region between *traW, traU* and *trbC*. Similarly, the antibiotic resistance region primer set (5′-CGTGCTCGGTCTGTCTTGTGTTTC-3′ and 5′-GCCTGCGCTCAAACGGACATT-3′) was used for amplification of the region between *cmlA1*, *aadA2*, and *aph(3″)-1b*. PCR products were subsequently assessed by sequencing (The Centre for Applied Genomics (TCAG) Facility, the Hospital for Sick Children) to confirm the presence of the associated region of interest.

To further investigate the antibiotic resistance gene region of p1AB5075 in *A. baumannii* AB046 transconjugants, two additional sets of diagnostic primers were used. One set (5′-TGTTCGGTTCGTAAACTGTAATGCAAGTAG-3′ and 5′-GCTGAATTGTGCTC-GCTGTCGTAC-3′) was used for amplification of the region that encompasses *ant(2″)-Ia* and *cmlA1*. The second set (5′-GACATAAGCCTGTTCGGTTGGTAAGC-3′ and 5′-TTACCGATTACGCCATTTTCTGACGT-3′) was used to amplify of the region between ABUW_4053, *bla*_GES-11_ and *aac(6′)-Ib10*. All PCR products were subsequently analysed by agarose gel electrophoresis.

### 2.4. Phylogenetic Analyses

Phylogenetic analyses were performed with host strains that were capable of plasmid acquisition using a previously established MLST approach [56]. Sequence data was extracted from full or partial genome data from either the NCBI database (*A. baumannii* AB5075-UW, Accession CP008706.1; *A. baumannii* ATCC 17978, Accession CP000521.1; *A. baumannii* AB046, Accession CP037872.1; *A. baumannii* AB048, Accession CP037870.1; *A. baumannii* AB053, Accession CP037869.1; *A. nosocomialis* DSM 102856, project KB849239.1; *A. seifertii* CIP 110471, project NZ_APOO00000000.1) or the ATCC database (*A. haemolyticus* ATCC 17906, *A. baylyi* ATCC 33305). Concatenated sequences were used for phylogenetic tree construction as previously described [58] using MrBayes v3.2 [59], with 200,000 generations sampled every 100 generations using a gamma distribution model and invariant class. Phylogenetic tree visualization was performed with Interactive Tree Of Life (iTOL) [60].

### 2.5. Minimum Inhibitory Concentration (MIC) Assays

To assess antibiotic susceptibility, minimum inhibitory concentration (MIC) assays were performed in 96-well microtiter plates. The broth microdilution method was used, and assays were performed according to CSLI guidelines [61]. As a culture medium, cation-adjusted Mueller–Hinton Broth (BD) was used, and microtitre plates were incubated without shaking at 37 °C.

### 2.6. Antibiotic Inactivation Assays

Antibiotic inactivation assays were performed on cefotaxime-resistant strains using a previously established method [62]. Liquid cultures supplemented with antibiotic (cefotaxime (20 μg/mL)) were inoculated with donor or transconjugant strains and incubated overnight at the appropriate temperature. Following centrifugation, culture supernatants were collected and used in disk diffusion assays, using *Micrococcus luteus* GDW1580 as a susceptible test organism. Inocula were prepared using the direct colony suspension method to the 0.5 McFarland standard, and supernatants were subsequently spotted on sterile filter disks. TSA plates with the susceptible test organism were incubated at 30 °C for 2 days, where the absence of a zone of inhibition was suggestive of antibiotic inactivation.

## 3. Results

### 3.1. Conjugative Transfer of T4SS-Encoding Plasmid to Environmental A. baumannii Strains

The XDR plasmid p1AB5075 from clinical isolate *A. baumannii* AB5075-UW (ST-1) harbours eleven resistance determinants spanning five different antimicrobial classes and is a member of a large group of at least 120 plasmids encoding an F-type T4SS gene cluster [37]. Previously, p1AB5075 was used as representative member of this group and was observed to transfer to clinically derived strain *A. baumannii* ATCC 17978 (ST-437) [37]. To investigate the capacity for conjugative transfer of this XDR plasmid to genetically divergent *A. baumannii* isolated from more diverse origins (i.e., environmental ecosystems), we examined a series of recipient strains previously isolated from water systems in Canada [52]. These isolates were genetically classified as ST-1039, 2250, and 2251 [52].

Conjugative transfer assays suggested that three of the five environmental *A. baumannii* strains served as successful recipients of p1AB5075 from donor *A. baumannii* AB5075-UW (Table 1 and Table S1). Conjugation frequencies were observed up to 5.8 × 10^−5^, with *A. baumannii* AB046 (ST-2250), demonstrating the highest frequency of transfer.

**Figure 1:**
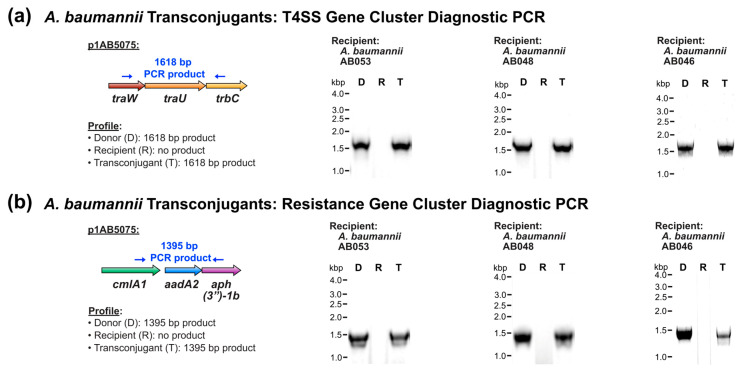
Conjugative transfer of the XDR plasmid p1AB5075 to environmental *A. baumannii* strains. (**a**,**b**) Diagnostic PCR analysis of the p1AB5075 T4SS-encoding gene cluster (**a**) and antibiotic resistance gene cluster (**b**).

In addition to verifying the genetic background of candidate transconjugants, the transfer of p1AB5075 was examined by diagnostic PCR and subsequent sequencing. In all candidate transconjugants examined, the presence of both the T4SS-encoding gene cluster and the antibiotic resistance gene cluster was detected (Figure 1a and Figure 1b, respectively). This demonstrates the potential for self-transmissibility to both more genetically divergent *A. baumannii* strains as well as strains of environmental origin.

### 3.2. Conjugative Transfer of T4SS-Encoding Plasmid to More Genetically Divergent Acinetobacter

Previous work established that the large group of F-type T4SS-encoding plasmids also included members from several non-*baumannii* species of *Acinetobacter* [37]. Given this observation, we examined the potential for the transfer of p1AB5075 to a collection of more genetically divergent *Acinetobacter* of both clinical and environmental origin. In particular, recipient strains included species that phylogenetically grouped within the ACB complex (*A. nosocomialis*, *A. seifertii*) and outside the ACB complex within one of two other subclades (*A. haemolyticus*, *A. junii*, and *A. baylyi*) [63]. Conjugative transfer assays suggested that four of the five strains were successful recipients of p1AB5075 (Table 2 and Table S2), with conjugation frequencies up to 5.6 × 10^−2^ with *A. nosocomialis* DSM 102856. These transfer frequencies are consistent with the range observed for *Acinetobacter* plasmids [44,64,65].

**Figure 2:**
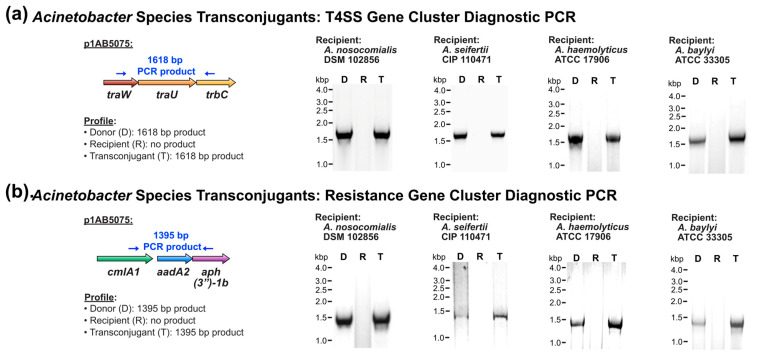
Conjugative transfer of the XDR plasmid p1AB5075 to more genetically divergent *Acinetobacter* species. (**a**,**b**) Diagnostic PCR analysis of the p1AB5075 T4SS-encoding gene cluster (**a**) and antibiotic resistance gene cluster (**b**).

Subsequent to verifying the genetic background of candidate transconjugants, the presence of plasmid p1AB5075 in transconjugants was assessed by diagnostic PCR and sequencing. In all the transconjugants examined, the T4SS-encoding gene cluster and the antibiotic resistance gene cluster in p1AB5075 were detected (Figure 2a and Figure 2b, respectively). Collectively, this suggests that the family of T4SS-encoding plasmids represented by XDR p1AB5075 has the potential for transmissibility to genetically diverse *Acinetobacter* strains of both clinical and environmental origin.

### 3.3. Antibiotic Susceptibility of Transconjugant Acinetobacter Strains

The plasmid p1AB5075 harbours determinants capable of conferring resistance to the aminoglycoside antibiotics kanamycin and tobramycin and the β-lactam cefotaxime [37]. Transconjugants were examined for antibiotic susceptibility by minimum inhibitory concentration (MIC) assay. Assessment of environmental *A. baumannii* strains showed that transconjugants for *A. baumannii* AB048 and AB053 exhibited decreased sensitivity to kanamycin, tobramycin, and cefotaxime compared to the associated recipients (Table 3). Similarly, transconjugants for *A. baumannii* AB046 also demonstrated decreased sensitivity to kanamycin relative to the recipient strain (Table 3). However, despite confirmation of the presence of p1AB5075 in *A. baumannii* AB046 transconjugants (Figure 1), at the phenotypic level, differences in susceptibility to tobramycin and cefotaxime were not observed compared to the recipient strain (Table 3). All transconjugants demonstrated susceptibility to ciprofloxacin that was characteristic of the respective recipient strains (Table 3).

**Figure 3:**
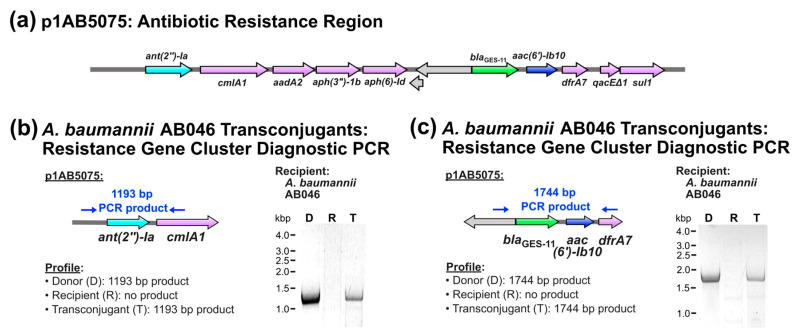
Assessment of p1AB5075 antibiotic resistance gene region in *A. baumannii* AB046 transconjugants. (**a**) The antibiotic resistance gene region of p1AB5075. Antibiotic resistance genes are shown in colour and genes not associated with antibiotic resistance are shown in grey. The resistance genes of interest, *ant(2″)-Ia*, *aac(6′)-Ib10*, and *bla*_GES-11_, are shown in cyan, light green, and blue, respectively, while all other resistance genes are denoted in light purple. (**b**,**c**) Diagnostic PCR analysis of p1AB5075 antibiotic resistance gene cluster. (**b**) Region containing *ant(2″)-Ia*. (**c**) Region containing *bla*_GES-11_ and *aac(6′)-Ib10*.

Transconjugants for *A. baumannii* AB046 demonstrated decreased susceptibility to kanamycin compared to the recipient strain but no susceptibility differences for tobramycin and cefotaxime (Table 3), despite confirmation of the presence of p1AB5075 (Figure 1). This pattern of susceptibility was reproducibly observed with multiple transconjugants per biological replicate assay and across multiple conjugation assays. Plasmid p1AB5075 contains a 9.6 kb region that harbours ten of the plasmid’s eleven antibiotic resistance determinants (Figure 3a). Among these are the determinants that are capable of conferring resistance to tobramycin (*ant(2″)-Ia* and *aac(6′)-Ib10*) [66,67] and cefotaxime (*bla*_GES-11_) [50]. To assess whether plasmid truncations occurred in these regions of p1AB5075 in *A. baumannii* AB046 transconjugants, targeted diagnostic PCR assays were performed. In all candidate transconjugants examined, both the *ant(2″)-Ia*-containing region and the *bla*_GES-11_ and *aac(6′)-Ib10*-containing region were detected (Figure 3b and Figure 3c, respectively). This suggests that large genetic rearrangements may not be responsible for the inability of *A. baumannii* AB046 transconjugants to confer resistance to tobramycin and cefotaxime.

Likewise, the antibiotic susceptibility of transconjugants of the more genetically divergent *Acinetobacter* species were examined via MIC assay. All transconjugants were capable of growth in higher levels of kanamycin, tobramycin, and cefotaxime compared to their respective recipient strains (Table 4). Also consistent with plasmid transfer, transconjugants retained sensitivity to ciprofloxacin, with levels comparable to that of the recipient (Table 4). Similarly, consistent with plasmid transfer, *A. baumannii* and more genetically divergent *Acinetobacter* transconjugants demonstrated resistance to trimethoprim and sulfamethoxazole (Table S3). Collectively, this demonstrates that, with the exception of strain *A. baumannii* AB046, successful transfer of the T4SS-encoding plasmid resulted in decreased susceptibility to the clinically important antibiotics cefotaxime and tobramycin.

### 3.4. Cefotaxime Inactivation by Transconjugant Acinetobacter Strains

**Figure 4:**
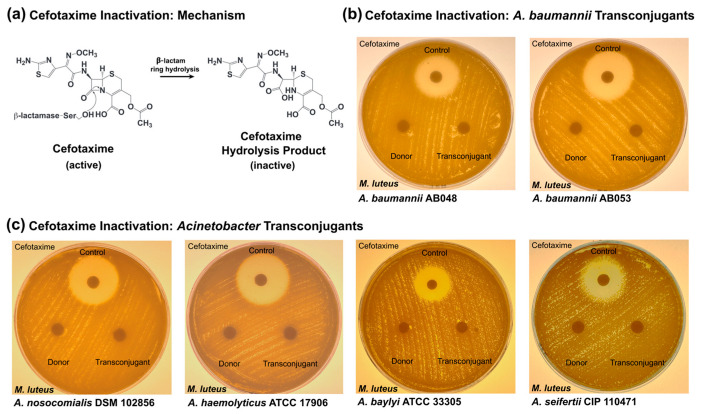
Cefotaxime inactivation by *Acinetobacter* transconjugants harbouring XDR plasmid p1AB5075. (**a**) Schematic diagram of cefotaxime inactivation. Plasmid p1AB5075 harbours the β-lactamase-encoding gene *bla*_GES-11_. Class A β-lactamases like Bla_GES-11_ use an active site serine residue to catalyse a ring-opening reaction of the β-lactam antibiotic. Due to the removal of the β-lactam ring, the key structural reactive centre of the antibiotic, the resulting product lacks antimicrobial activity. (**b**,**c**) Antibiotic disk diffusion assays. Resistant *Acinetobacter* strains were grown in the presence of cefotaxime, and culture supernatants were assessed by disk diffusion assay against the indicator organism *M. luteus*. Assays were performed in parallel with the donor strain *A. baumannii* AB5075-UW. (**b**) *A. baumannii* strains: *A. baumannii* AB048 transconjugant and *A. baumannii* AB053 transconjugant. (**c**) More genetically divergent *Acinetobacter* strains: *A. nosocomialis* DSM 102856 transconjugant, *A. seifertii* CIP 110471 transconjugant, *A. haemolyticus* ATCC 17906 transconjugant, and *A. baylyi* ATCC 33305 transconjugant.

β-Lactamases represent a large and genetically diverse class of antibiotic resistance enzymes that function by hydrolysing the β-lactam ring structure that is essential for antimicrobial activity [68]. Plasmid p1AB5075 harbours the β-lactamase gene *bla*_GES-11_ [50], encoding a β-lactamase capable of inactivating carbapenems, cephalosporins, and other β-lactam antibiotics (Figure 4a). Antibiotic inactivation assays [62] were performed with β-lactam-resistant *Acinetobacter* strains. Culture media from cefotaxime resistant *A. baumannii* transconjugants of environmental origin grown in the presence of antibiotic did not contain active β-lactam (Figure 4b), consistent with this mechanism of antibiotic resistance. Likewise, the more genetically divergent *Acinetobacter* transconjugants also demonstrated the capacity to functionally inactivate cefotaxime (Figure 4c). Collectively, this demonstrates that conjugative transfer of the T4SS-encoding plasmid is capable of conferring β-lactamase activity to more genetically divergent recipient strains both inside and outside the *A. baumannii* species, including strains of both clinical and environmental origin.

## 4. Discussion

**Figure 5:**
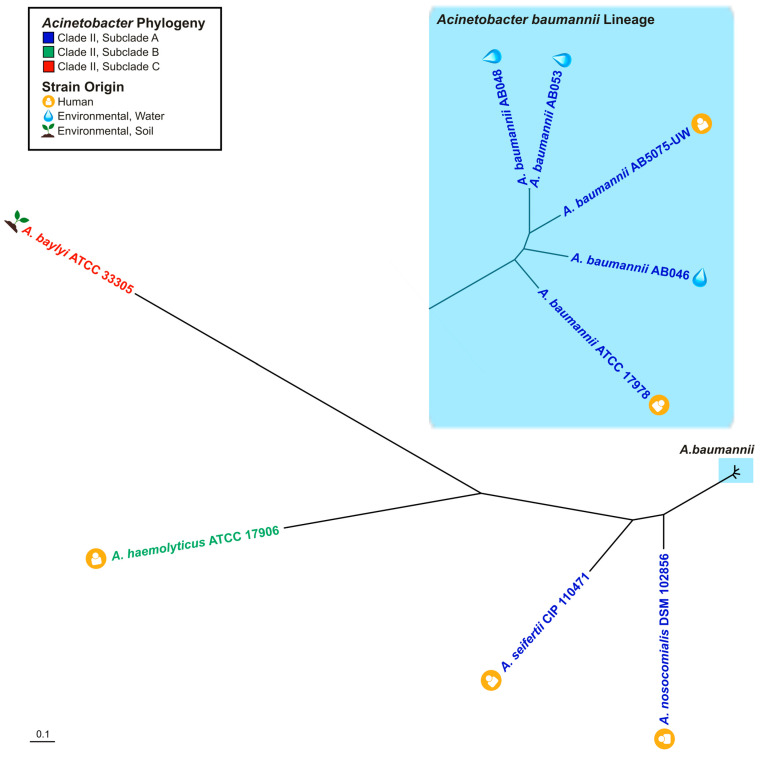
Host strain diversity for T4SS-encoding plasmid member p1AB5075. Phylogenetic tree of recipient *Acinetobacter* strains for p1AB5075. Strain names are colour-coded based on the established clade [63] and subclade shown in Table 2, as reflected in the legend. Strain origin is indicated based on the symbols shown in the legend. The inset shown in light blue represents the *A. baumannii* lineage of *Acinetobacter* hosts.

The WHO has described antibiotic resistance as among of the greatest threats to human health and, together with the Centers for Disease Control and Prevention (CDC), have cited *Acinetobacter baumannii* as an MDR pathogen at the highest levels of priority for the research community. Given this pathogen’s exceptional capacity to acquire genetic material, including antibiotic resistance determinants [4], insight into the mechanisms that promote the spread of resistance is of great importance. In this study, the candidate host range of an XDR member of a large class of *Acinetobacter* T4SS-encoding plasmids [37] was examined. The finding that p1AB5075 demonstrates the capacity to transfer to *A. baumannii* strains and *Acinetobacter* species spanning several subclades of the phylogenetic tree (Figure 5) provides new insight into the potential host spectrum within the *Acinetobacter* genus. Among non-*baumannii Acinetobacter* species with the capacity to serve as plasmid recipients were species of clinical importance, including several species considered to be emerging human pathogens [21,22]. Given these observations and that a member of this plasmid family was previously documented to exist in a clinical strain of *Klebsiella pneumoniae* [37], it is possible that the plasmid has the capacity to transfer beyond the subclades tested, including to multiple genera of Gammaproteobacteria.

It is well recognized that a One Health Approach to understanding infectious diseases is needed to address the challenge of antimicrobial resistance [34]. In the context of *Acinetobacter*, surveillance endeavours in environmental- and agricultural-associated settings are increasingly identifying environmental *Acinetobacter* as a reservoir of importance for antibiotic resistance [27,69,70,71]. Of importance to this study were recipient strains from environmental sources, which included water and soil ecosystems. The observation that environmental strains of *Acinetobacter*, including *A. baumannii*, demonstrated the capacity to serve as recipients for the representative XDR member of T4SS-encoding plasmids (Figure 5) suggests that this plasmid family may have the capacity to spread to *Acinetobacter* in environmental reservoirs as well.

In the context of conjugation assays, it is interesting to note that conjugation frequencies did not always correlate with the genetic relatedness between the donor and recipient. For example, selected non-*baumannii* recipients demonstrated higher conjugation frequencies than *A. baumannii* recipients. This observation was not unexpected, given the diverse ecological niches from which the recipients were derived. It is well documented that a myriad of other factors can impact differences in conjugation efficiencies between recipients, including the conjugation methodological approach (e.g., liquid vs. solid medium) [72]. Other potential factors of relevance include differing nutrient needs, native growth rates, native moisture content needs, and the presence or absence of certain small molecules [73,74,75,76,77,78,79].

In the context of native conjugation in natural environments, other complex factors may also impact conjugation efficiency. For example, while conjugation assays reflect plasmid transfer under simplistic conditions (i.e., one donor and one recipient), native microbial populations are more complex and variable in composition. As such, factors such as native population composition, population density, and nutrient availability may impact plasmid transfer [73,74,75,80]. In addition, because *Acinetobacter* strains have the capacity to form biofilms, additional considerations may impact plasmid transfer, such as nutrient availability, local oxygen concentration, and level of bacterial dormancy [75,81,82].

Another notable observation was that while transconjugants of *A. baumannii* AB046 demonstrated decreased susceptibility to kanamycin compared to the recipient, they showed equivalent susceptibility levels for cefotaxime and tobramycin. This observation was exclusively observed for *A. baumannii* AB046 transconjugants and was consistently observed with multiple transconjugants per biological replicate assay and across multiple conjugation assays. While diagnostic PCR assays did not suggest large genetic rearrangements in the regions of the resistance genes responsible (Figure 4b,c), other possibilities may account for this interesting observation. One such possibility is that *A. baumannii* AB046 transconjugants demonstrate differential levels of antibiotic resistance gene expression compared to *A. baumannii* AB5075-UW. Variations in resistance gene expression levels between donor and transconjugants has been previously documented [83].

Increasing rates of infection by β-lactam-resistant *Acinetobacter* strains have been acknowledged by both the WHO and CDC as a growing challenge for the healthcare community [1]. Of importance to this research, the p1AB5075-containing group of T4SS-encoding plasmids represents a large reservoir of β-lactam resistance genes [37], with 42% of resistance determinants encoding resistance to β-lactams [37]. The finding that even more genetically divergent *Acinetobacter* transconjugants harbouring p1AB5075 exhibited both resistance to the clinically important β-lactam cefotaxime and the ability to inactivate the drug suggests that this plasmid family has the capacity to be phenotypically functional and therefore potentially problematic if spread among strains of clinical importance.

Recently, a large-scale survey and genetic analysis of *A. baumannii* plasmids proposed a classification system for plasmid lineages [48]. Of importance, the lineage harbouring F-type T4SS gene clusters, including p1AB5075 and pACICU2, represented the largest class of plasmids identified in the study [48]. Also of importance, genetic analyses of these T4SS-encoding plasmids identified other plasmid-encoded genes that may contribute to pathogenesis or survival in the host, such as universal stress protein-encoding genes and copper homeostasis gene clusters [37]. Given our observations of transfer to *Acinetobacter* spanning several subclades of the phylogenetic tree, including non-*baumannii Acinetobacter* species of clinical importance and several emerging human pathogens, coupled with potential contributions to pathogen fitness and the vast reservoir of antibiotic resistance genes encoded within, this research highlights a group of *Acinetobacter* plasmids that could be of importance for future surveillance efforts.

## Figures and Tables

**Table 1:** Conjugative transfer of multidrug-resistance plasmid p1AB5075 from *A. baumannii* AB5075-UW to environmental *A. baumannii* stains.

Recipient Strain	MLST Sequence Type ^1^	Strain Origin ^2^	Plasmid Transfer	Conjugation Frequency ^3^
*A. baumannii* AB053	1039	E, stream	Positive	L ^5^
*A. baumannii* AB052	unknown ^4^	E, stream	Negative	N/A ^6^
*A. baumannii* AB048	1039	E, river	Positive	L ^5^
*A. baumannii* AB047	2251	E, river	Negative	N/A ^6^
*A. baumannii* AB046	2250	E, river	Positive	5.8 × 10^−5^

^1^ Pasteur MLST sequence type. ^2^ E, environmental. ^3^ Mean transconjugants per recipient cell. ^4^ Sequence type unknown due to partial genome sequencing data. ^5^ L, transconjugants were obtained with a conjugation frequency too low to quantify. In all instances, multiple transconjugants were obtained in each experiment, and the observation was reproducible across three biological replicate experiments. ^6^ N/A, not applicable.

**Table 2:** Conjugative transfer of multidrug-resistance plasmid p1AB5075 from *A. baumannii* AB5075-UW to more genetically divergent *Acinetobacter* species.

Recipient Strain	Phylogenetic Designation (Clade, Subclade (If Applicable)) ^1^	Strain Origin ^2^	Plasmid Transfer	Conjugation Frequency ^3^
*A. nosocomialis* DSM 102856	II, A	C, human	Positive	5.6 × 10^−2^
*A. seifertii* CIP 110471	II, A	C, human	Positive	L ^4^
*A. haemolyticus* ATCC 17906	II, B	C, human	Positive	4.7 × 10^−3^
*A. junii* ATCC 17908	II, B	C, human	Negative	N/A ^5^
*A. baylyi* ATCC 33305	II, C	E, soil	Positive	3.6 × 10^−9^

^1^ Phylogenetic classification of clades based on Almeida, O.G.G., et al. [63]. Note that the subclades of Clade II are denoted as Subclades A, B, and C, where *A. baumannii* belongs to Subclade A. ^2^ C, clinical, E, environmental. ^3^ Mean transconjugants per recipient cell. ^4^ L, transconjugants were obtained with a conjugation frequency too low to quantify. In all instances, multiple transconjugants were obtained in each experiment, and the observation was reproducible across three biological replicate experiments. ^5^ N/A, not applicable.

**Table 3:** Minimum inhibitory concentrations (MICs) of antibiotics against *A. baumannii* strains.

Strain ^1^	MIC (μg/mL)			
	Kanamycin	Tobramycin	Cefotaxime	Ciprofloxacin
*A. baumannii* AB5075-UW	>256	64	>256	64
*A. baumannii* AB053-R	2	≤1	16	≤1
*A. baumannii* AB053-T	>256	16	>256	≤1
*A. baumannii* AB048-R	2	≤1	16	≤1
*A. baumannii* AB048-T	>256	8	>256	≤1
*A. baumannii* AB046-R	2	≤1	8	≤1
*A. baumannii* AB046-T	>256	≤1	8	≤1

^1^ R, recipient, T, transconjugant.

**Table 4:** Minimum inhibitory concentrations (MICs) of antibiotics against more genetically divergent *Acinetobacter* species.

Strain	MIC (μg/mL)			
	Kanamycin	Tobramycin	Cefotaxime	Ciprofloxacin
*A. baumannii* AB5075-UW	>256	64	>256	64
*A. nosocomialis* DSM 102856-R	8	2	16	≤1
*A. nosocomialis* DSM 102856-T	>256	32	>256	≤1
*A. seifertii* CIP 110471-R	2	≤1	8	≤1
*A. seifertii* CIP 110471-T	>256	16	>256	≤1
*A. haemolyticus* ATCC 17906-R	16	16	8	≤1
*A. haemolyticus* ATCC 17906-T	>256	64	>256	≤1
*A. baylyi* ATCC 33305-R	≤1	≤1	4	≤1
*A. baylyi* ATCC 33305-T	>256	16	>256	≤1

## Data Availability

The original contributions presented in this study are included in the article/Supplementary Materials. Further inquiries can be directed to the corresponding author.

## Supplementary Materials

The following supporting information can be downloaded at: https://www.mdpi.com/article/10.3390/pathogens14060606/s1, Table S1: Conjugal transfer of multidrug resistance plasmid p1AB5075 from *A. baumannii* AB5075-UW to environmental *A. baumannii* stains; Table S2: Conjugal transfer of multidrug resistance plasmid p1AB5075 from *A. baumannii* AB5075-UW to more genetically divergent *Acinetobacter* species; Table S3: Antibiotic susceptibility testing of *A. baumannii* and more genetically divergent *Acinetobacter* transconjugants.
