# Supplementary material for: Characterization of the Diversity in Host Range of an Extensively Drug-Resistant (XDR) Type IV Secretion System-Encoding Plasmid in Acinetobacter
Source: Pathogens. 2025 Jun 19;14(6):606. doi: 10.3390/pathogens14060606 (PMC12196012; doi:10.3390/pathogens14060606)
Supplement: Supplementary file 1 [file pathogens-14-00606-s001.zip › pathogens-3607530-supplementary.pdf]

SUPPLEMENTARY MATERIAL

Characterization of the Diversity in Host Range Of an Extensively Drug-resistant (XDR)Type IV Secretion System-encoding Plasmid in *Acinetobacter*

Kailey Martz[1,2], Dalya Alomar[1,2†], Marisha Karim[1,2†], Sara Knezevic[1,2] and Vanessa M. D'Costa[1,2*]

[1] Department of Biochemistry, Microbiology and Immunology, University of Ottawa, Ottawa, Ontario, Canada, K1H 8M5
[2] Centre for Infection, Immunity and Inflammation, University of Ottawa, Ottawa, Canada, K1H 8M5

* Corresponding author:
Vanessa M. D'Costa
Department of Biochemistry, Microbiology and Immunology
University of Ottawa
451 Smyth Road
Ottawa, Ontario, Canada
K1H 8M5
Tel: (613)-562-2800 ext 4541
E-mail: vdcosta@uottawa.ca

† These authors contributed equally

SUPPLEMENTARY TABLES

Supplementary Table 1. Conjugal Transfer of Multidrug Resistance Plasmid p1AB5075 from *A. baumannii* AB5075-UW to Environmental *A. baumannii* Stains

Recipient Strain	Biological Replicate Experiment	Conjugation Frequency[1]
A. baumannii AB053	1	L[2]
	2	L[2]
	3	L[2]
A. baumannii AB048	1	L[2]
	2	4.8×10^{-8}
	3	L[2]
A. baumannii AB046	1	1.4×10^{-4}
	2	2.6×10^{-5}
	3	1.1×10^{-5}

[1] Transconjugants per recipient cell

[2] L, transconjugants were obtained with conjugation frequency too low to quantify. In all instances, multiple transconjugants were obtained in each experiment.

Supplementary Table 2. Conjugal Transfer of Multidrug Resistance Plasmid p1AB5075 from *A. baumannii* AB5075-UW to More Genetically Divergent *Acinetobacter* Species

Recipient Strain	Biological Replicate Experiment	Conjugation Frequency[1]
A. nosocomialis DSM 102856	1	9.7×10^{-2}
	2	1.8×10^{-2}
	3	5.2×10^{-2}
A. seifertii CIP 110471	1	L[2]
	2	L[2]
	3	L[2]
A. haemolyticus ATCC 17906	1	4.1×10^{-3}
	2	5.2×10^{-3}
	3	4.8×10^{-3}
A. baylyi ATCC 33305	1	4.6×10^{-10}
	2	6.9×10^{-9}
	3	3.5×10^{-9}

[1] Transconjugants per recipient cell

[2] L, transconjugants were obtained with conjugation frequency too low to quantify. In all instances, multiple transconjugants were obtained in each experiment.

Supplementary Table 3. Antibiotic Susceptibility Testing of *A. baumannii* and More Genetically Divergent *Acinetobacter* Transconjugants

Strain[1]	Trimethoprim[2]	Sulfamethoxazole[2]
A. baumannii AB5075-UW	R	R
A. baumannii AB053-R	S	S
A. baumannii AB053-T	R	R
A. baumannii AB048-R	S	S
A. baumannii AB048-T	R	R
A. baumannii AB046-R	S	S
A. baumannii AB046-T	R	R
A. nosocomialis DSM 102856-R	S	S
A. nosocomialis DSM 102856-T	R	R
A. seifertii CIP 110471-R	S	S
A. seifertii CIP 110471-T	R	R
A. haemolyticus ATCC 17906-R	S	S
A. haemolyticus ATCC 17906-T	R	R
A. baylyi ATCC 33305-R	S	S
A. baylyi ATCC 33305-T	R	R

[1] R, recipient, T, transconjugant.

[2] R, resistant at 64 μg/mL, S, sensitive at 64 μg/mL
